# Prognostic significance of branched-chain amino acid transferase 1 and CD133 in triple-negative breast cancer

**DOI:** 10.1186/s12885-020-07070-2

**Published:** 2020-06-22

**Authors:** Yu Song, Bin Zhao, Yali Xu, Xinyu Ren, Yan Lin, Liangrui Zhou, Qiang Sun

**Affiliations:** 1 Department of Breast Surgery, Peking Union Medical College Hospital, Chinese Academy of Medical Sciences and Peking Union Medical College, No. 1 Shuaifuyuan, Dongcheng District, Beijing, 100730 China; 2 Department of Pathology, Peking Union Medical College Hospital, Chinese Academy of Medical Science and Peking Union Medical College, Beijing, China

**Keywords:** Triple-negative breast cancer, BCAT1, CD133, Prognosis, Biomarker

## Abstract

**Background:**

Previous studies have shown that branched-chain amino acid transferase 1 (BCAT1) is associated with tumour progression in triple-negative breast cancer (TNBC). Furthermore, CD133 has emerged as a novel cancer stem cell marker for indicating tumour progression. However, the prognostic significance of these two markers remains to be verified. This study was conducted to investigate the correlation between BCAT1 and CD133 expression and clinicopathological features, as well as the prognosis of patients with TNBC.

**Methods:**

The study cohort included 291 patients with TNBC. Tissue microarrays were constructed for both cancer and normal tissues. The expression of BCAT1 and CD133 was detected by immunohistochemical staining, and the levels were evaluated using an H-scoring system. Cut-off points for BCAT1 and CD133 expression were determined using receiver operating characteristic curves.

**Results:**

The median follow-up time for the study participants was 68.73 months (range: 1.37–103.6 months). The 5-year disease-free survival (DFS) and overall survival (OS) rates of the 291 patients with TNBC were 72.51 and 82.47%, respectively. Higher levels of BCAT1 and CD133 expression independently indicated shorter DFS and OS. High levels of both BCAT1 and CD133 expression were detected in 36 (12.37%) patients, who had significantly shorter DFS and OS (both *P* < 0.001) compared to other patients.

**Conclusion:**

BCAT1 and CD133 can be considered as biomarkers with prognostic significance for TNBC.

## Background

Triple-negative breast cancer (TNBC) accounts for approximately 15% of all breast cancer cases [1]. TNBC is characterised by negative expression of the oestrogen receptor (ER), progesterone receptor (PR), and human epidermal growth factor receptor 2 (HER2). TNBC is more prone to hematogenous than to lymphatic metastasis, resulting in a higher incidence of visceral metastases, particularly to the lungs and brain [2, 3]. Basal-like breast cancer (BLBC) accounts for 80–90% of TNBC cases and is characterised by high levels of expression levels of basal cell-specific proteins [4]. The pathogenesis of BLBC overlaps with that of TNBC, and BLBC is generally considered as an important subtype of TNBC [5, 6]. Statistics have confirmed that compared with other breast cancer subtypes, TNBC has a higher recurrence rate and worse prognosis [7–9]. The development of novel prognostic indicators and possible therapeutic targets is thus imperative for improving the diagnostic and treatment options for TNBC.

Branched-chain amino acid transferase 1 (BCAT1) is a key cytoplasmic enzyme that decomposes branched-chain amino acids, including leucine, valine, and isoleucine, which are crucial signalling molecules in the PI3K/AKT/mTOR signalling network and are involved in the metabolism of glucose, lipids, and proteins [10]. During the breakdown process, BCAT1 provides energy for the synthesis of mitochondrial ATP and various macromolecules [11]. BCAT1 levels have been positively associated with tumour progression and an unfavourable prognosis in many malignancies, including gastric cancer, glioma, hepatic cancer, and nasopharyngeal carcinoma [12–15]. Compared with those in normal breast tissue, BCAT1 levels have been shown to be elevated in various breast cancer tissues, including in invasive carcinoma, intraductal carcinoma, and lobular carcinoma [16, 17]. However, the association between BCAT1 expression and TNBC prognosis requires confirmation in more comprehensive studies.

Cancer stem cells (CSCs) are a limited subpopulation of cells with stem cell-like properties. CD133, also known as prominin 1 (PROM1), is a transmembrane protein expressed on the surface of hematopoietic stem cells and widely recognised as a CSC marker. CD133 is reportedly associated with a cancer-related signalling system and promotes tumour migration, invasion, and progression [18–20]. Studies have suggested a prognostic relationship between CD133 expression and melanoma, hepatocellular cancer, prostate cancer, and glioma [21–24]. In breast cancer, CD133 expression indicates CSC-like characteristics and tumourigenic effects, with increased tumour self-renewal, high tumour cell proliferation, and drug resistance, confirming the prognostic significance of CD133 [25, 26]. However, the relationship between the expression of CD133, as a CSC marker and potential prognostic indicator, and TNBC prognosis should be verified in a larger study cohort.

Discovering novel prognostic factors and potential therapeutic targets for TNBC has been a major focus area of clinical research. Herein, we aimed to investigate the prognostic significance of BCAT1 and CD133 and their relationships with the clinicopathological factors of TNBC.

## Methods

### Patients and follow-up information

**Fig. 1:**
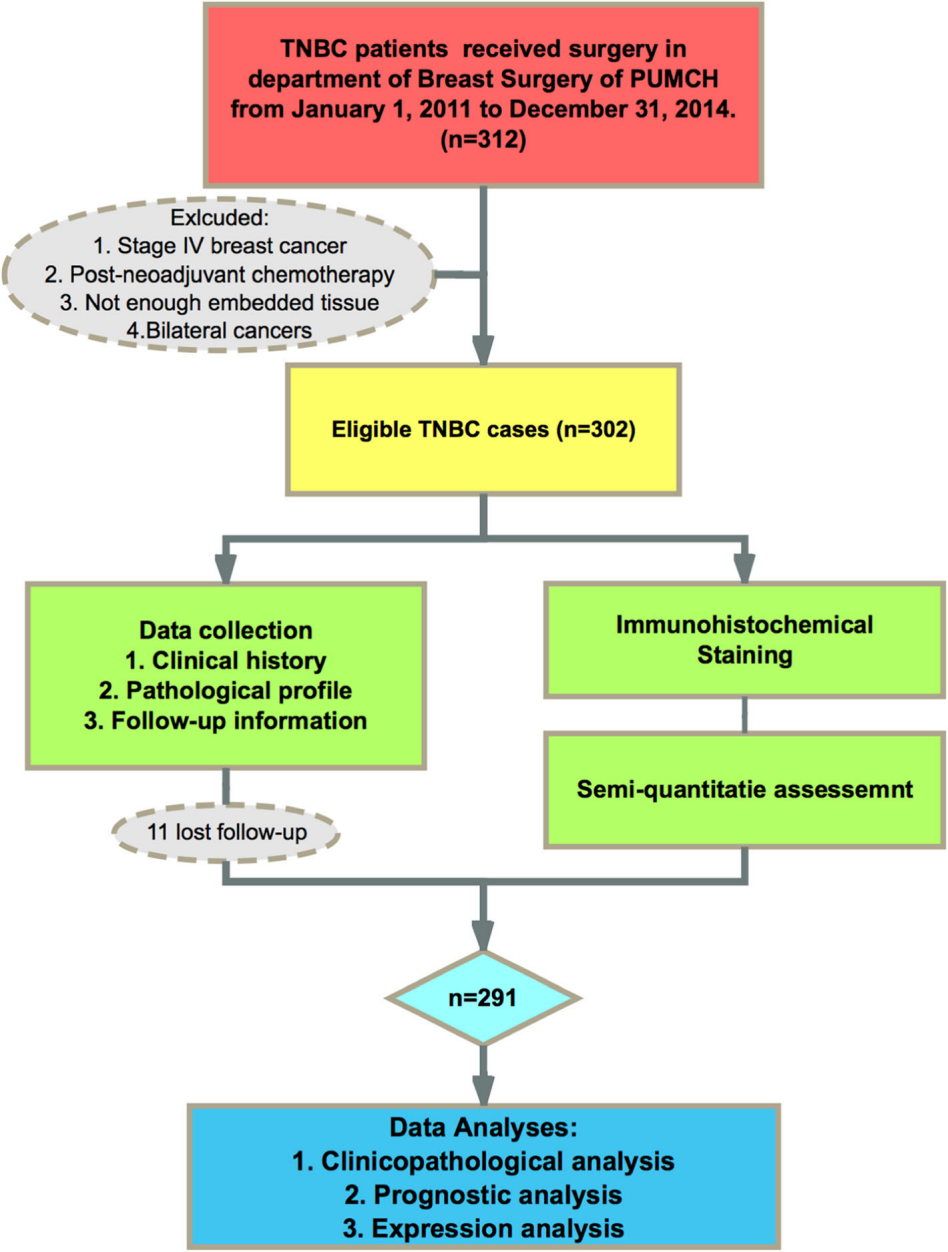
Flowchart of the study. A cohort of 291 patients with triple-negative breast cancer (TNBC) was enrolled, with complete clinical profiles and follow-up information

A cohort of 302 patients with stage I to III primary unilateral untreated TNBC was included in this study. All the patients received a curative surgery at the Department of Breast Surgery of the Peking Union Medical College Hospital between 1 January 2011 and 31 December 2014, followed by adjuvant chemotherapy or radiotherapy, which was administered according to the National Comprehensive Cancer Network guidelines [27]. All the clinical and pathological information of the patients were obtained from their medical records and pathology reports. Clinical parameters such as age, gender, menstrual status, family history of breast cancer, and medical history of adjuvant treatment and pathological parameters such as tumour size, histological tumour grade, lymphvascular invasion, tumour infiltrating lymphocytes, axillary lymph node status, and pathological stage based on the 8th edition of the AJCC staging system were considered for the study. Cases with insufficient paraffin-embedded tumour samples, incomplete clinical or pathological profiles, unclear follow-up information, or stage IV TNBC, as well as those treated with neoadjuvant chemotherapy (NAC) were excluded from the study, which resulted in a final cohort of 291 patients, as shown in the study flow diagram (Fig. 1).

The follow-up period of this retrospective study was from the date of surgery until 31 March 2019, during which the survival information of the patients was monitored. Disease-free survival (DFS) was defined as the time from diagnosis to the first relapse of the disease (local, regional, distant, or contralateral breast cancer), and OS was defined as the time from diagnosis to death from any cause. Relapsed disease and metastasis were verified by diagnostic imaging and pathology during follow-up examinations.

This study was approved by the Institutional Review Board of the Peking Union Medical College Hospital. Written informed consent was obtained from all participants.

### Tissue microarray (TMA) construction

The paraffin-embedded tissue blocks collected included cancer and normal tissues. All tissues were fixed in 10% neutral-buffered formalin immediately after surgical resection and promptly embedded in paraffin. Haematoxylin and eosin (H&E)-stained slides of each block were reviewed by two experienced pathologists to confirm the diagnosis and to mark the most representative areas of the tumours. TMAs were constructed by two pathologists as per previous studies [28–30], using an automated tissue arrayer (Aphelys MiniCore 3; Mitogen, Harpenden, UK). Each case was sampled twice, according to the previous marks, to obtain 2.0-mm punch cores, and each TMA contained 100 cores from 50 cases. After reheating for proper fusion fixation, all TMAs were stored for sectioning and further examination.

### Immunohistochemical (IHC) staining and analysis

Serial sections (3–4 μm) were mounted on adhesive slides. IHC staining was performed using a Ventana Benchmark XT autostainer and standard autostaining protocols, as per the manufacturer’s instructions (Ventana Medical Systems, Inc., Tucson, AZ, USA), and the antibodies used in the protocol were listed in Table 1. For positive and negative controls, the manufacturer-recommended control tissue and isotype antibody were used, respectively. All slides were quality controlled by an experienced technician and reviewed by two pathologists.

**Table 1:** Immunohistochemical staining standards for the pathological biomarkers

Antibody	Clone	Dilution	Source	Positive style	Positive control	Cutoff values (%)	Antigen retrieval	Incubation
Erα	Rabbit monoclonal	Prediluted	Epitomics	Nuclear staining	Breast cancer	≥1	100 °C, 30 min	37 °C, 32 min
PR	Rabbit monoclonal	Prediluted	Epitomics	Nuclear staining	Breast cancer	≥1	100 °C, 30 min	37 °C, 32 min
HER-2	Rabbit monoclonal	Prediluted	Ventana	Membrane staining	Breast cancer	≥3+ or FISH+	100 °C, 30 min	37 °C, 32 min
CK5/6	Mouse monoclonal	Prediluted	Dako	Membrane staining and/or cytoplasmic staining	Mesothelioma	≥5	100 °C, 31 min	37 °C, 32 min
EGFR	Rabbit monoclonal	Prediluted	Ventana	Membrane staining and/or cytoplasmic staining	Skin	≥25	100 °C, 32 min	37 °C, 32 min
P53	Mouse monoclonal	Prediluted	MXB	Nuclear staining	Colon adenocarcinoma	≥5	100 °C, 33 min	37 °C, 32 min
Ki-67^a^	Mouse monoclonal	Prediluted	ZSGB-BIO	Nuclear staining	Breast cancer	≥14	100 °C, 34 min	37 °C, 32 min
BCAT1	Rabbit polyclonal	1:100	Absin	Cytoplasmic staining	hepatocellular cancer	≥90	100 °C, 35 min	37 °C, 32 min
CD133	Rabbit Monoclonal	1:400	CST	Membrane staining and/or cytoplasmic staining	Lymphnodes	≥95	100 °C, 36 min	37 °C, 32 min

^a^Ki-67 index threshold of 14% was chosen according to the St. Gallen Consensus 2013

The TMA IHC slides were independently reviewed by two experienced pathologists, who were blinded to the experimental conditions. In the absence of a univariate evaluation method of BCAT1 and CD133 expression using IHC staining, a semiquantitative H-scoring system was used, with both the staining intensity and the percentage of positive cancer cells taken into consideration [31–33]. The staining intensity was evaluated using four grades: 0, negative; 1, weak; 2, moderate; and 3, strong. The percentage of positive cancer cells was calculated for representative areas. The staining intensity and average percentage of positive cells were evaluated for 10 independent high-magnification fields. The final weighted-expression H-scores, ranging from 0 to 300, were obtained by multiplying the staining intensity by the percentage of positive cells. The final score for each case was provided by two pathologists, who reassessed any inconsistent cases and agreed on the final scores. The total-expression H-scores for BCAT1 and CD133 were presented as continuous variables for correlation analysis.

The expression of other biomarkers was evaluated according to standard protocols (Table 1). A five-biomarker immunopanel (ER, PR, HER2, CK5/6, and EGFR) was used to classify TNBC cases as basal-like (ER^−^/PR^−^/HER2^−^, with EGFR^+^ and/or CK5/6^+^) or non-basal-like (ER^−^/PR^−^/HER2^−^/EGFR^−^/CK5/6^−^). The expression of these biomarkers was determined according to the St. Gallen Consensus 2013 [34]. TILs were independently evaluated by two pathologists in a blinded manner, according to international recommendations [35].

### Bioinformatic validation of gene expression in TNBC

For the gene expression analysis and validation of BCAT1 and CD133 in patients with TNBC that underwent NAC, or that of different TNBC subtypes, the public microarray GEO datasets were retrieved (https://www.ncbi.nlm.nih.gov/gds/) and analysed with GraphPad Prism 7.0a (GraphPad Software, San Diego, CA, USA). For analysis of NAC responses, the GEO datasets GSE25055, GSE25066, GSE41998 and GSE106977 were collected. For analysis of gene expression across multiple TNBC subtypes, the GEO datasets GSE86945 and GSE86946 were collected.

### Statistical analysis

Statistical analysis was carried out using the SPSS 25.0 software (SPSS, Inc., Chicago, IL, USA). The Kaplan–Meier method was used to estimate survival of patients. Qualitative variables were compared using the chi-squared test. Cox proportional hazard models was used to estimate hazard ratio and 95% confidence interval for clinicopathological variables associated with DFS and OS. The association was first assessed by univariate log-rank test, and variables with *P* values < 0.05 were entered into the multivariate Cox regression analysis with the forward stepwise regression method. The discriminatory power of prognostic factors was assessed using receiver operating characteristic (ROC) curve analysis to identify the optimal value of a continuous variable and to differentiate between the probability of survival and death. A two-sided *P*-value of less than 0.05 was considered statistically significant in all tests.

## Results

### Patients’ clinicopathological characteristics and baseline information

The clinicopathological characteristics of the 291 cases with TNBC were analysed using the follow-up information (Table 2). The median age in the recurrent group was 49.5 (range: 25 to 79) years, and that in the non-recurrent group was 49 (range: 25 to 77) years. Medium- to high-histological-grade invasive breast ductal carcinoma was mostly encountered (290/291, 99.66%). Among the cases with adequate pathological indications, 51.54% (150/291) received chemotherapy, and 38.49% (112/291) received radiation treatment. The median overall follow-up time was 68.73 (range: 1.37 to 103.6) months. The estimated 5-year DFS rate for the cohort was 72.2% (standard error: 2.3%), and the 5-year OS rate was 81.6% (standard error: 2.3%).

**Table 2:** Baseline clinicopathological characteristics of patients

Clinicopahtological criteria	No. of patients	Patients with recurrence *n* = 82	Patients without recurrence *n* = 209	*P*
Age at diagnosis				0.602
≤ 49	154	41 (50.00%)	113 (54.07%)	
> 49	137	41 (50.00%)	96 (45.93%)	
Menopausal status				0.897
Pre-menopause	148	41 (50.00%)	107 (51.2%)	
Post-menopause	143	41 (50.00%)	102 (48.8%)	
Histological grade				0.393
I/II	85	27 (32.93%)	58 (27.75%)	
III	206	55 (67.07%)	151 (72.25%)	
Tumor size				0.015
pT1	136	31 (37.80%)	105 (50.24%)	
pT2	139	42 (51.22%)	97 (46.41%)	
pT3	12	8 (9.76%)	4 (1.91%)	
pT4	4	1 (1.22%)	3 (1.44%)	
Nodal status				0.000
negative	165	34 (41.46%)	131 (62.68%)	
1–3 nodes	63	15 (18.29%)	48 (22.97%)	
4–9 nodes	27	10 (12.2%)	17 (8.13%)	
≥ 10 nodes	36	23 (28.05%)	13 (6.22%)	
Stage				0.000
I	92	15 (18.29%)	77 (36.84%)	
II	134	33 (40.24%)	101 (48.33%)	
III	65	34 (41.37%)	31 (14.83%)	
Basal-like phenotype				0.850
Negative	40	12 (14.63%)	28 (13.4%)	
Positive	251	70 (85.37%)	181 (86.6%)	
Ki67 index^a^				0.030
≤ 14	30	14 (17.07%)	16 (7.66%)	
> 14	261	68 (82.93%)	193 (92.34%)	
TIL				0.002
Low	153	55 (67.07%)	98 (46.89%)	
High	138	27 (32.93%)	111 (53.11%)	
Family History				1.000
Negative	275	78 (95.12%)	197 (94.26%)	
Positive	16	4 (4.88%)	12 (5.74%)	
Chemotherapy				0.297
Negative	32	6 (7.32%)	26 (12.44%)	
Positive	259	76 (92.68%)	183 (87.56%)	
Radiotherapy				0.016
Negative	179	41 (50.00%)	138 (66.03%)	
Positive	112	41 (50.00%)	71 (33.97%)	
LVI				0.188
Negative	270	74 (90.23%)	196 (93.78%)	
Positive	21	8 (9.77%)	13 (6.22%)	
CD133				0.002
H score (mean ± SE)		98.85 ± 8.534	68.74 ± 4.039	
Low expression	174	37 (45.12%)	137 (65.55%)	
High expression	117	45 (54.88%)	72 (34.45%)	
BCAT1				0.000
H score (mean ± SE)		101.46 ± 7.259	72.80 ± 3.758	
Low expression	205	44 (53.66%)	161 (77.03%)	
High expression	86	38 (46.34%)	48 (22.97%)	

*TIL* tumor infiltrating lymphocytes, *LVI* lymphovascular invasion, *SE* standard error. ^a^Ki-67 index threshold of 14% was chosen according to the St. Gallen Consensus 2013

### High expression levels of BCAT1 and CD133 indicated an unfavourable prognosis

**Fig. 2:**
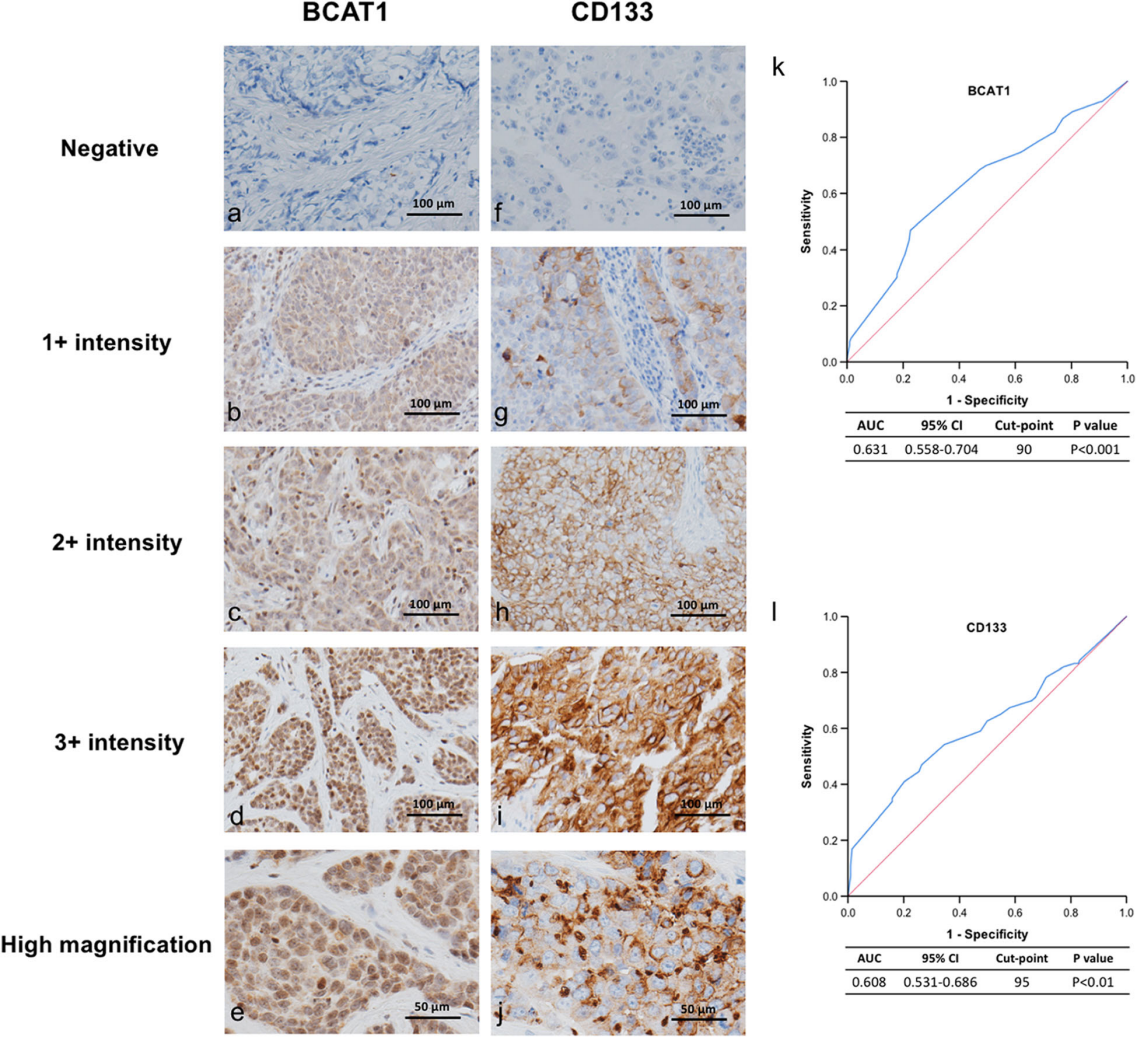
Immunohistochemical staining patterns and cut-points for BCAT1 (**a**–**e**, and **k**) and CD133 (**f**–**j**, and **l**) in triple-negative breast-cancer tissues

**Fig. 3:**
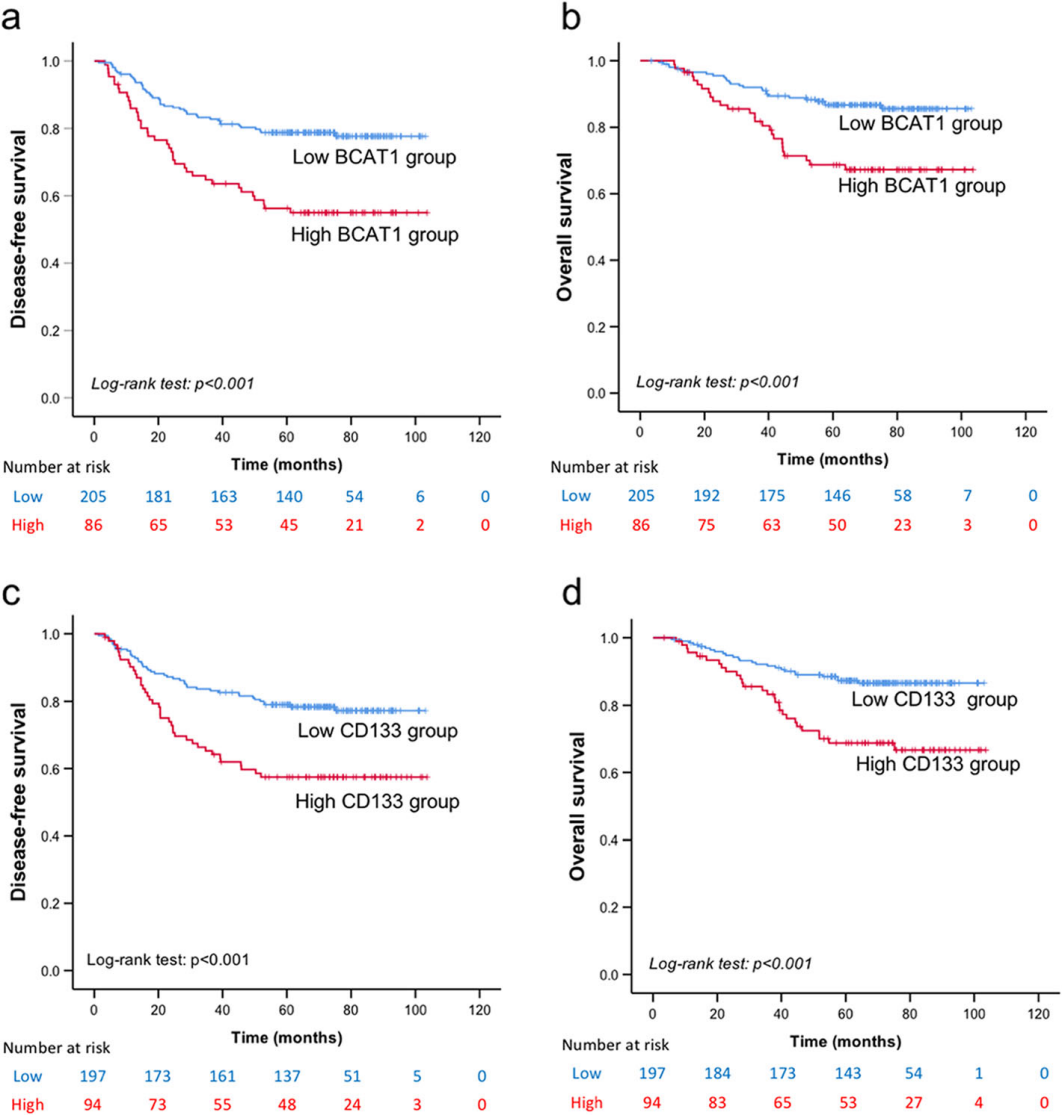
Kaplan–Meier survival analysis based on BCAT1 and CD133 expression. Disease-free survival in (**a**) low- and high-BCAT1 groups and (**c**) low- and high-CD133 groups. Overall survival in (**b**) low- and high-BCAT1 groups and (**d**) low- and high-CD133 groups. *P* < 0.001 in all cases

BCAT1 staining was mainly observed in the cytoplasm, and CD133 staining was mainly displayed in the membrane (Fig. 2a-e, and f-j). The H-score for BCAT1 expression was significantly higher among the patients with recurrence than among those without recurrence (*P* < 0.001), and CD133 showed a similar pattern (*P* = 0.002) (Table 2). Cut-point values were determined using ROC curve analysis, and the cut-point scores for BCAT1 and CD133 expression were 90 and 95, respectively (Fig. 2k and l). Based on these data, the cohort was divided into low- and high-expression groups. The estimated 5-year DFS rates in the low- and high-BCAT1 expression groups were 78.8 and 56.2%, and the estimated 5-year OS rates were 86.8 and 68.7%, respectively. The estimated 5-year DFS rates in the low- and high-CD133 expression groups were 79.0 and 57.5%, and the estimated 5-year OS rates were 87.4 and 68.7%, respectively (Table 3). Significantly shorter (all *P* < 0.001) DFS and OS were found in the high-BCAT1 expression group (Fig. 3a and b) and in the high-CD133 expression group (Fig. 3c and d).

**Table 3:** Correlation of TNBC prognosis with BCAT1 and CD133 expressions

Variable	No. of Patient	DFS		*P*	OS		*P*
		No. of event	Estimated 5-year surviving rate (standard error)		No. of event	Estimated 5-year surviving rate (standard error)	
cohort	291	83	72.2% (2.6%)	–	53	81.6% (2.3%)	–
BCAT1 expression							
Low	205	44	78.8% (2.9%)	*P* < 0.001	27	86.8% (2.4%)	*P* < 0.001
High	86	39	56.3% (5.4%)		26	68.7% (5.2%)	
CD133 expression							
Low	197	44	79.0% (2.9%)	*P* < 0.001	25	87.4% (2.4%)	*P* < 0.001
High	94	39	57.5% (5.2%)		28	68.7% (5.0%)	
BCAT1 + CD133 expression							
Both-low expression	147	26	82.9% (3.1%)	*P* < 0.001	13	90.8% (2.4%)	*P* < 0.001
mixed expression	108	35	68.0% (4.5%)		27	76.8% (4.2%)	
both-high expression	36	21	39.9% (8.3%)		14	55.0% (9.1%)

**Fig. 4:**
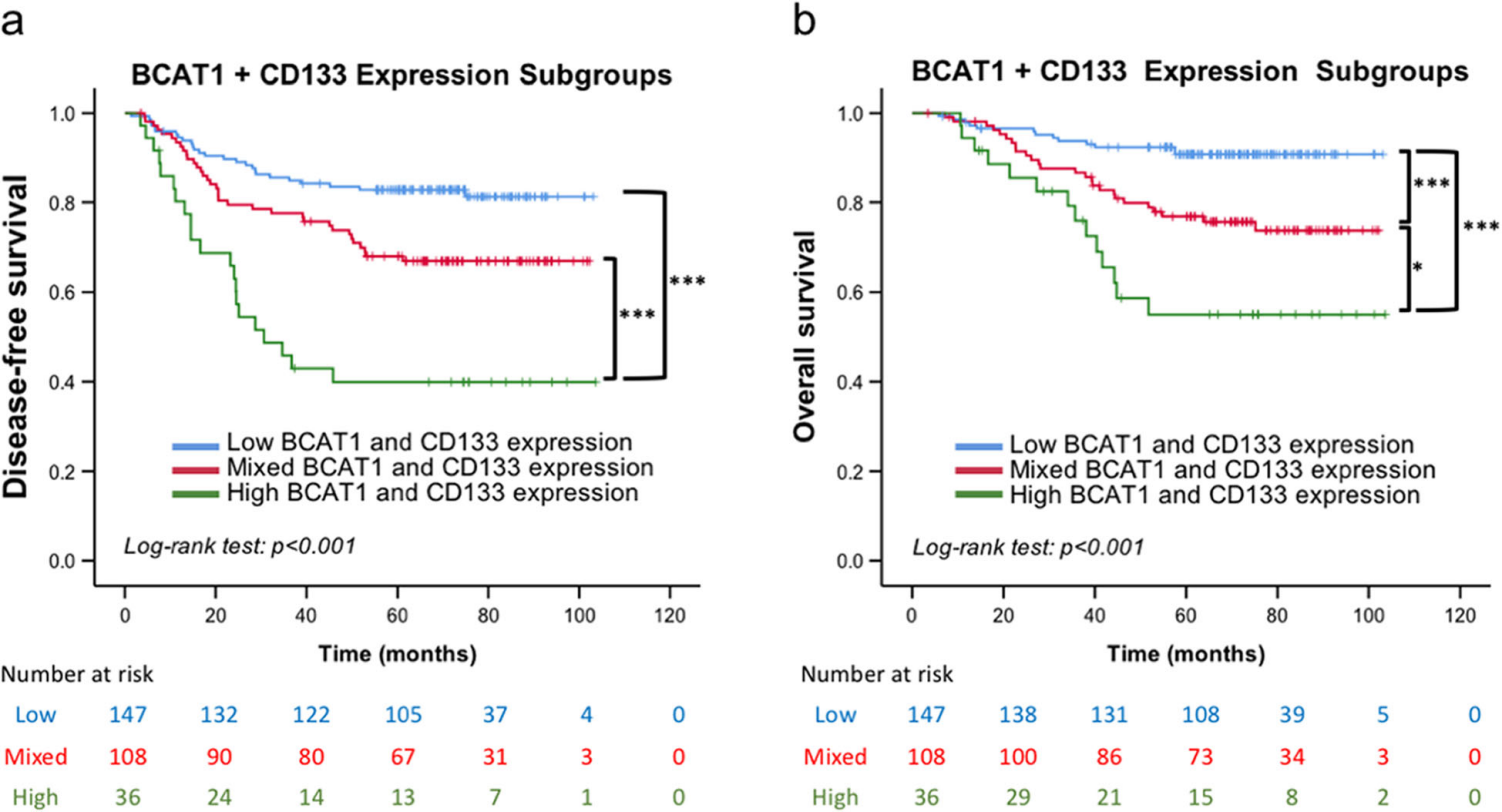
Kaplan–Meier survival analysis based on a combined biomarker set (BCAT1 and CD133). Disease-free survival (**a**) and overall survival (**b**) in double-low-expression, mixed-expression, and double-high-expression groups. *P < 0.05, ***P < 0.001

When BCAT1 and CD133 expression was used as a combined biomarker set, the cohort was divided into three groups: a double-low-expression group (147/291, 50.52%), mixed-expression group (108/291, 37.11%), and double-high-expression group (36/291, 12.37%). The survival rate decreased as both biomarkers were found to be highly expressed (Fig. 4a and b). Compared with those in the double-low- or mixed-expression group, a significantly shorter DFS and OS were found when either one or both of the biomarkers were highly expressed (Fig. 4a and b).

### Correlation between clinicopathological factors and BCAT1 and CD133 expression

BCAT1 expression was found to be associated with the TIL level, histological grade, and the Ki67 index but not with criteria such as the age, lymphovascular invasion, tumour size, axillary lymph node status, and pathological stage (Table 4). A higher level of TILs was found to be correlated with low BCAT1 expression (*P* = 0.007). High BCAT1 expression was associated with a higher histological grade (*P* = 0.009) and a higher Ki67 index (*P* = 0.036). CD133 expression showed no correlation with the clinicopathological criteria (Table 4).

**Table 4:** Relation between CD133 and BCAT1 markers with clinical pathological features in TNBC patients

Clinicopathological criteria	No. of patients	BCAT1 n (%)		*P*	CD133 n (%)		*P*
		Low expression	High expression		Low expression	High expression	
Age at diagnosis				0.25			0.802
≤ 49	154	113 (73.37%)	41 (26.63%)		103 (66.88%)	51 (33.12%)	
> 49	137	92 (67.15%)	45 (32.85%)		94 (68.61%)	43 (31.39%)	
Menopausal status				0.522			0.453
Pre-menopause	148	107 (72.29%)	41 (27.71%)		97 (65.54%)	51 (34.46%)	
Post-menopause	143	98 (68.53%)	45 (31.47%)		100 (69.93%)	43 (30.07%)	
Family History				0.785			1
Negative	275	193 (70.18%)	82 (29.82%)		186 (67.63%)	89 (32.37%)	
Positive	16	12 (75%)	4 (25%)		11 (68.75%)	5 (31.25%)	
Histological grade				0.009			0.743
I	1	0 (0%)	1 (100%)		1 (100%)	0 (0%)	
II	84	50 (59.52%)	34 (40.48%)		58 (69.04%)	26 (30.96%)	
III	206	155 (75.24%)	51 (24.76%)		138 (66.99%)	68 (33.01%)	
Tumor size				0.322			0.483
pT1	136	89 (65.44%)	47 (34.56%)		91 (66.91%)	45 (33.09%)	
pT2	139	105 (75.53%)	34 (24.47%)		95 (68.34%)	44 (31.66%)	
pT3	12	8 (66.66%)	4 (33.34%)		7 (58.33%)	5 (41.67%)	
pT4	4	3 (75%)	1 (25%)		4 (100%)	0 (0%)	
Nodal status				0.624			0.453
Negative	165	119 (72.12%)	46 (27.88%)		114 (69.09%)	51 (30.91%)	
1–3 nodes	63	45 (71.42%)	18 (28.58%)		40 (63.49%)	23 (36.51%)	
4–9 nodes	27	19 (70.37%)	8 (29.63%)		21 (77.77%)	6 (22.23%)	
≥ 9 nodes	36	22 (61.11%)	14 (38.89%)		22 (61.11%)	14 (38.89%)	
TNM Stage				0.584			0.907
I	92	64 (69.56%)	28 (30.44%)		63 (68.47%)	29 (31.53%)	
II	134	98 (73.13%)	36 (26.87%)		89 (66.41%)	45 (33.59%)	
III	65	43 (66.15%)	22 (33.85%)		45 (69.23%)	20 (30.77%)	
Basal phenotype				0.853			0.044
Negative	40	29 (72.5%)	11 (27.5%)		33 (82.5%)	7 (17.5%)	
Positive	251	176 (70.11%)	75 (29.89%)		164 (65.33%)	87 (34.67%)	
Ki67 index^a^				0.036			0.84
≤ 14	30	16 (53.33%)	14 (46.67%)		21 (70%)	9 (30%)	
> 14	261	189 (72.41%)	72 (27.59%)		176 (67.43%)	85 (32.57%)	
TIL				0.007			0.315
Low	153	97 (63.39%)	56 (36.61%)		108 (70.58%)	45 (29.42%)	
High	138	108 (78.26%)	30 (21.74%)		89 (64.49%)	49 (35.51%)	
LVI				1			0.146
Negative	270	190 (70.37%)	80 (29.63%)		186 (68.88%)	84 (31.12%)	
Positive	21	15 (71.42%)	6 (28.58%)		11 (52.38%)	10 (47.62%)	
Chemotherapy				0.682			0.842
Negative	32	24 (75%)	8 (25%)		21 (65.62%)	11 (34.38%)	
Positive	259	181 (69.88%)	78 (30.12%)		176 (67.95%)	83 (32.05%)	
Radiotherapy				0.895			0.305
Negative	179	127 (70.94%)	52 (29.06%)		117 (65.36%)	62 (34.64%)	
Positive	112	78 (69.64%)	34 (30.36%)		80 (71.42%)	32 (28.58%)	

*TIL* tumor infiltrating lymphocytes, *LVI* lymphovascular invasion. ^a^Ki-67 index threshold of 14% was chosen according to the St. Gallen Consensus 2013

### Survival analysis

**Fig. 5:**
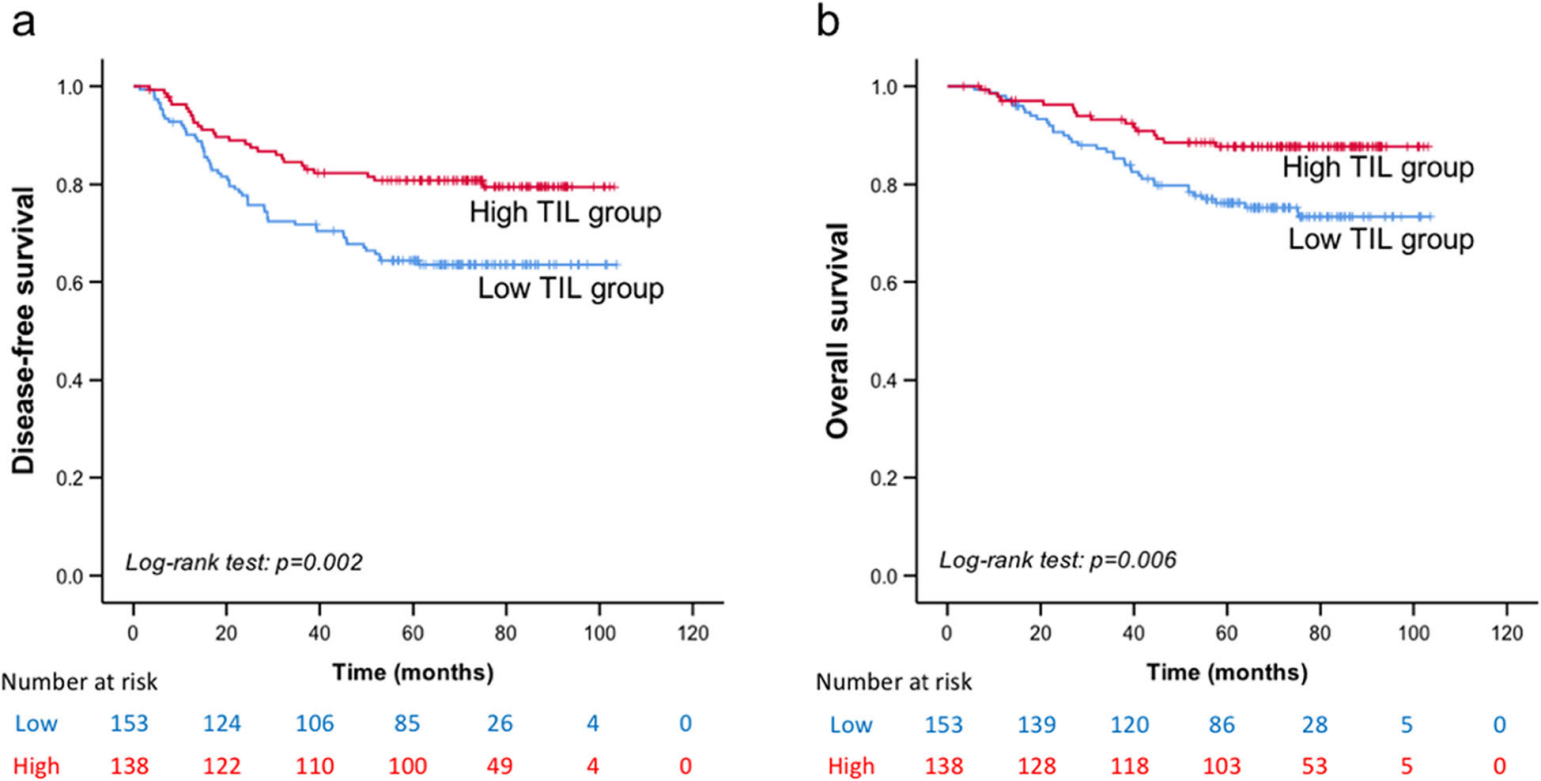
Kaplan–Meier survival analysis based on tumour-infiltrating lymphocyte (TIL) levels. Disease-free survival (**a**) and overall survival (**b**) of patients with high and low TIL levels. Both *P* < 0.01

**Fig. 6:**
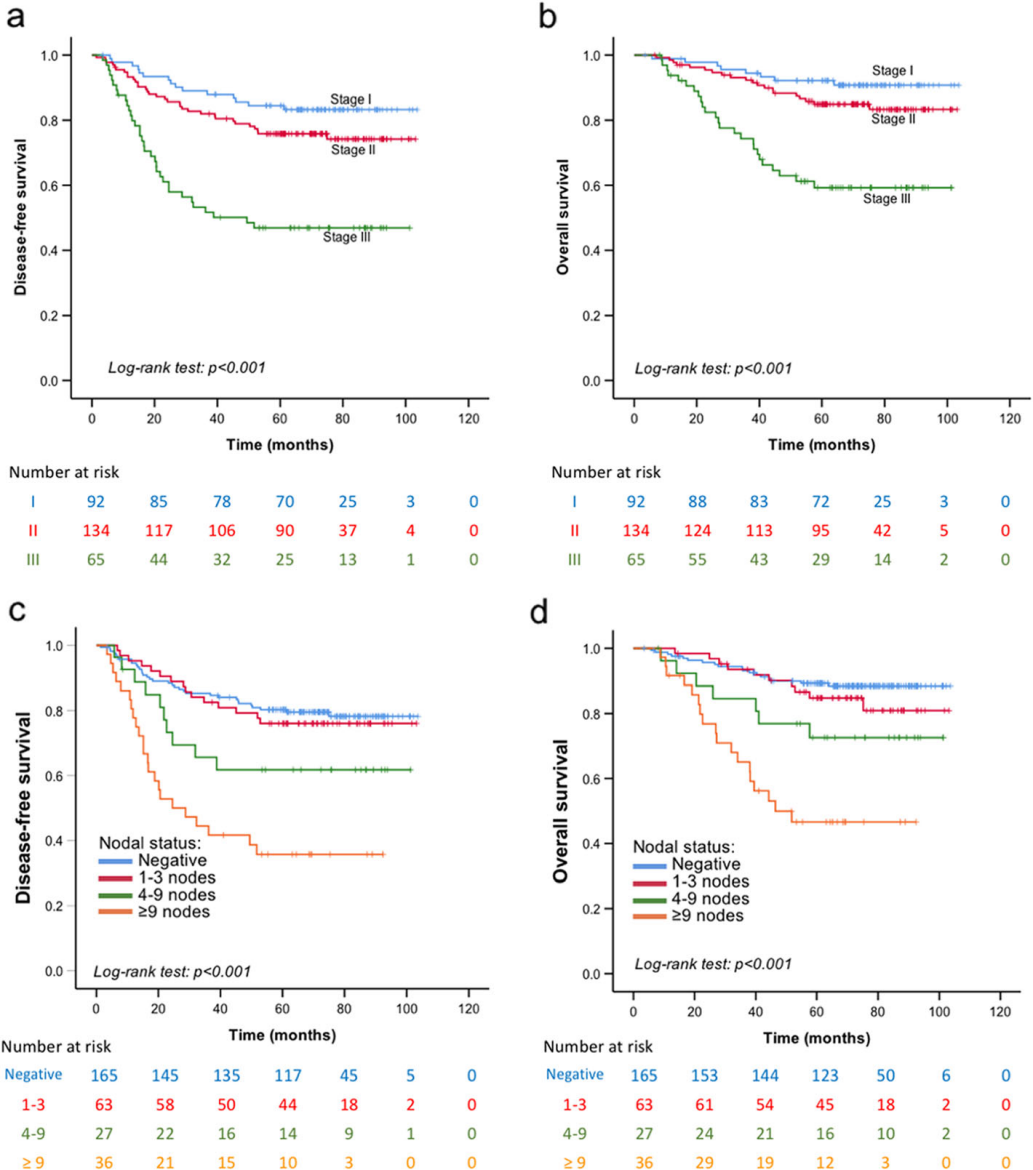
Kaplan–Meier survival analysis based on the tumour stage and nodal status. Disease-free survival in patients (**a**) with tumour TNM stages I–III and (**c**) different nodal statuses. Overall survival in patients (**b**) with tumour TNM stages I–III and (**d**) different nodal statuses. *P* < 0.001 in all cases

Univariate and multivariate Cox regression analyses were conducted for DFS (Table 5) and OS (Table 6). The independent predictors for both DFS and OS were the TIL level (*P* = 0.007 and *P* = 0.033, respectively), BCAT1 expression (*P* = 0.003 and *P* = 0.001, respectively), and CD133 expression (*P* < 0.001 and *P* = 0.001, respectively). Additionally, the tumour stage was an independent predictor for DFS (*P* < 0.001), and the nodal status was found to be independently correlated with OS (*P* < 0.001). The TIL level was found to be positively associated with TNBC prognosis, with prolonged DFS (*P* = 0.001; Fig. 5a) and OS (*P* = 0.007; Fig. 5b) observed in the high-TIL subgroup. The TNM stage (Fig. 6a and b) and nodal status (Fig. 6c and d) showed a negative association with DFS and OS.

**Table 5:** Univariate and multivariate Cox regression analysis of prognostic value of clinicopathological factors and BCAT1 and CD133 expression for DFS

Variable (DFS)	Univariate cox regression analysis	*P*	Multivariate cox regression analysis	*P*
	HR (95% CI)		HR (95% CI)	
Age at diagnosis		0.480		
≤ 49	1			
> 49	1.168 (0.759–1.796)			
Menopausal status		0.830		
Pre-menopause	1			
Post-menopause	1.048 (0.682–1.612)			
Histological grade		0.587		
I/II	1			
III	0.881 (0.556–1.394)			
Tumor size		0.001		0.136
pT1	1		NA	
pT2	1.443 (0.907–2.296)			
pT3/pT4	3.770 (1.793–7.930)			
Nodal status		0.000		0.112
negative	1		NA	
1–3 nodes	1.216 (0.671–2.203)			
4–9 nodes	2.060 (1.018–4.172)			
≥ 10 nodes	4.586 (2.692–7.812)			
TNM Stage		0.000		0.000
I	1		1	
II	1.638 (0.892–3.008)		1.744 (0.945–3.22)	
III	4.390 (2.388–8.071)		5.025 (2.722–9.278)	
Basal-like phenotype		0.773		
Negative	1			
Positive	0.914 (0.496–1.685)			
Ki67 index^a^		0.031		0.743
≤ 14	1		NA	
> 14	0.530 (0.298–0.943)			
TIL		0.004		0.007
Low	1		1	
High	0.507 (0.322–0.800)		0.525 (0.328–0.841)	
Family History		0.849		
Negative	1			
Positive	0.907 (0.332–2.478)			
Chemotherapy		0.199		
Negative	1			
Positive	1.724 (0.751–3.956)			
Radiotherapy		0.005		0.964
Negative	1		NA	
Positive	1.846 (1.200–2.840)			
LVI		0.190		
Negative	1			
Positive	1.630 (0.786–3.380)			
BCAT1		0.000		0.003
Low expression	1		1	
High expression	2.475 (1.608–3.810)		1.969 (1.259–3.079)	
CD133		0.000		0.000
Low expression	1		1	
High expression	2.154 (1.399–3.316)		2.42 (1.556–3.764)	

*TIL* tumor infiltrating lymphocytes, *LVI* lymphovascular invasion. ^a^Ki-67 index threshold of 14% was chosen according to the St. Gallen Consensus 2013. *NA* not applicable

**Table 6:** Univariate and multivariate Cox regression analysis of prognostic value of clinicopathological factors and BCAT1 and CD133 expression for OS

Variable (OS)	Univariate cox regression analysis	*P*	Multivariate cox regression analysis	*P*
	HR (95% CI)		HR (95% CI)	
Age at diagnosis		0.346		
≤ 49	1			
> 49	1.295 (0.755–2.221)			
Menopausal status		0.777		
Pre-menopause	1			
Post-menopause	1.081 (0.631–1.853)			
Histological grade		0.306		
I/II	1			
III	0.747 (0.426–1.309)			
Tumor size		0.004		0.188
pT1	1		NA	
pT2	1.362 (0.76–2.44)			
pT3/pT4	3.975 (1.677–9.422)			
Nodal status		0.000		0.000
negative	1		1	
1–3 nodes	1.451 (0.67–3.143)		1.434 (0.660–3.114)	
4–9 nodes	2.595 (1.084–6.215)		2.904 (1.198–7.044)	
≥ 10 nodes	6.487 (3.362–12.516)		5.780 (2.968–11.259)	
TNM Stage		0.000		0.349
I	1		NA	
II	1.794 (0.79–4.074)			
III	5.625 (2.535–12.483)			
Basal-like phenotype		0.911		
Negative	1			
Positive	1.046 (0.472–2.318)			
Ki67 index^a^		0.025		0.672
≤ 14	1		NA	
> 14	0.463 (0.233–0.922)			
TIL		0.006		0.033
Low	1		1	
High	0.449 (0.25–0.808)		0.519 (0.284–0.949)	
Family History		0.261		
Negative	1			
Positive	0.339 (0.047–2.452)			
Chemotherapy		0.345		
Negative	1			
Positive	1.626 (0.587–4.506)			
Radiotherapy		0.011		0.776
Negative	1		NA	
Positive	1.984 (1.156–3.403)			
LVI		0.089		
Negative	1			
Positive	2.06 (0.88–4.823)			
BCAT1		0.000		0.001
Low expression	1		1	
High expression	2.605 (1.519–4.467)		1.912 (1.094–3.342)	
CD133		0.000		0.001
Low expression	1		1	
High expression	2.703 (1.575–4.638)		2.554 (1.459–4.471)	

*TIL* tumor infiltrating lymphocytes, *LVI* lymphovascular invasion. ^a^Ki-67 index threshold of 14% was chosen according to the St. Gallen Consensus 2013. *NA* not applicable

### Bioinformatic validation

**Fig. 7:**
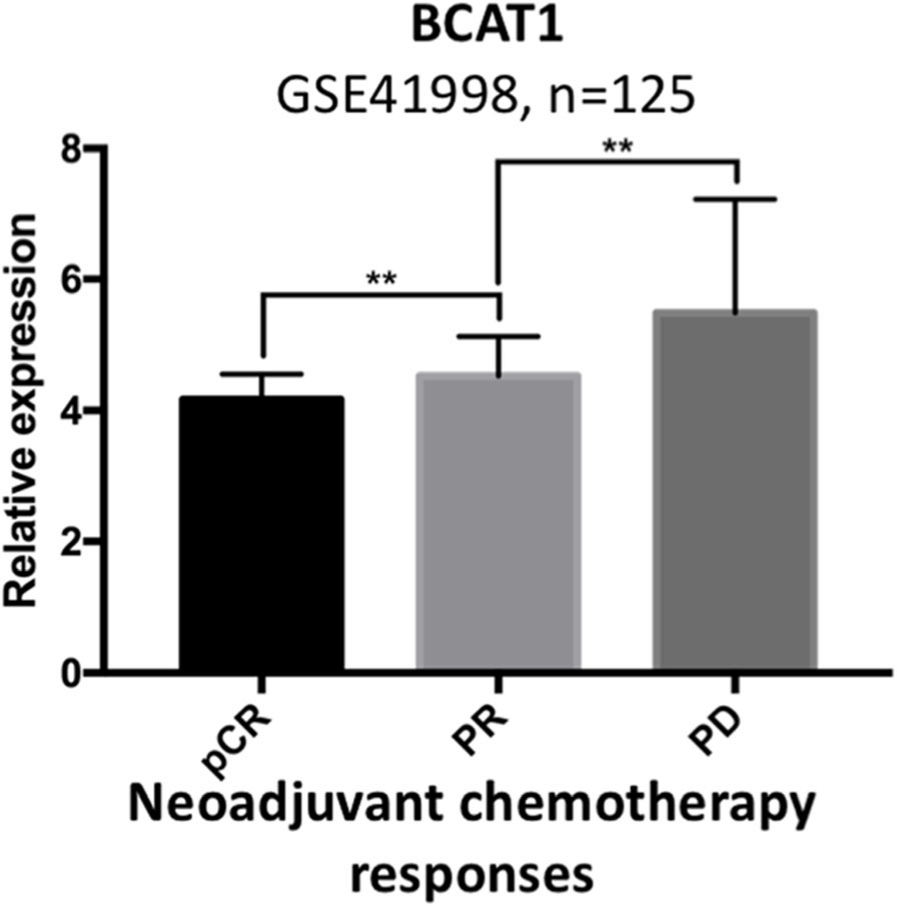
Bioinformatical analysis of BCAT1 expression of patients with TNBC treated with neoadjuvant chemotherapy. The neoadjuvant chemotherapy responses of patients with TNBC were retrieved and analyzed (GSE41998, *n* = 125). **P < 0.01

Analysis of public microarray data (GSE41998) of patients with TNBC who received NAC suggested that BCAT1 expression is correlated with different NAC responses including pathological complete remission (pCR), partial remission and progressed disease (*P* < 0.01). Higher levels of BCAT1 expression were detected in groups of patients with less favourable responses to NAC (Fig. 7).

**Fig. 8:**
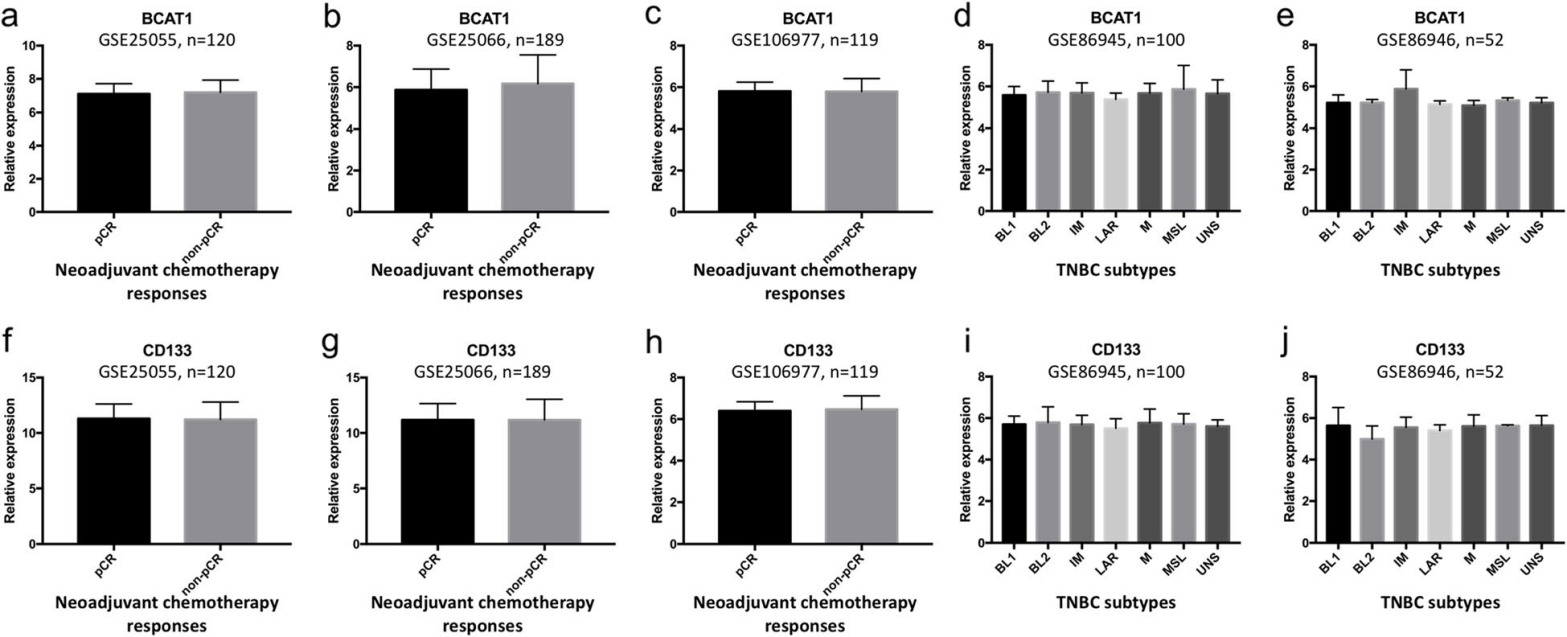
Bioinformatical analysis of biomarker expression in TNBC with neoadjuvant chemotherapy responses and multiple subtypes. No significant differences of BCAT1 or CD133 expression were detected among groups of different neoadjuvant chemotherapy responses (**a**-**c**, **f**-**h**) or different subtypes (**d**, **e**, **i** and **j**)

Furthermore, as for datasets (GSE25055, GSE25066 and GSE106977) with patients’ NAC responses marked as pCR and non-pCR, no significant differences were detected with BCAT1 or CD133 expression (Fig. 8a-c and f-h). As for datasets (GSE86945 and GSE86946) with TNBC subtype, no significant differences of BCAT1 or CD133 expression were detected among different TNBC subtypes groups (Fig. 8d, e, i and j).

## Discussion

In this study, the original cohort included 302 patients with TNBC with a median follow-up time of longer than 5 years. The expression levels of various biomarkers were evaluated semi-quantitatively, and the pattern of biomarker distribution was assessed, providing more vivid information for further validation of the prognostic significance of the target biomarkers. Several studies have examined prognostic biomarkers for TNBC; however, some of the supporting data are insufficient. A few studies have detected progress at the molecular and gene levels but not in terms of clinical application [36, 37]. The association of BCAT1 gene expression with that of ERα and tumour progression has been explored in cell culture and murine models with only limited samples from patients with TNBC and non-specific IHC staining [38]. CD133, a CSC marker, is reportedly associated with breast cancer progression; however, additional data are needed to validate its prognostic significance in patients with TNBC [39].

A comprehensive statistical analysis was performed to assess the prognostic significance of BCAT1 and CD133 expression and the potentially related clinicopathological features. The findings of this study confirmed that BCAT1 and CD133 were independent predictors of TNBC prognosis. The relationships between BCAT1 and CD133 expression and clinical manifestations of TNBC, revealed in this study, were consistent with the data from some previous mechanistic studies. Thus, it has been shown that the expression of BCAT1, which is regulated by the DOT1L histone methyltransferase, promoted the migrations of breast cancer cells [36]. Another study has suggested that BCAT1 mainly promotes tumour proliferation by contributing to amino acid metabolism in glioma [13]. BCAT1 knockdown has been shown to lead to alpha-α-ketoglutarate accumulation and abrogation of the leukaemia-initiating potential, suggesting that the BCAA–BCAT1–alpha-α-ketoglutarate pathway as a therapeutic target for reducing leukaemia stem cell function in patients with acute myelogenous leukaemia [40]. CD133 has been reported to induce stem cell characteristics, and the level of CD133 expression is correlated with that of PLC-β2, the Ras/ERK pathway, and the Akt (PKB) survival pathway [19, 22, 41]. Regardless of the underlying mechanisms, BCAT1 and CD133 have been proposed to be involved in the tumour metabolism and progression, eventually leading to cancer recurrence and even death. The underlying mechanisms regulating tumour progression are complex, and further studies are necessary to validate these biomarkers as potential therapeutic targets.

In addition to prognostic indications, these biomarkers show clinical potential, including in personalised pathological assessment and precision medicine, is promising. Along with the use of the traditional prognostic indicators ER, PR, and HER2, the assessment of BCAT1 expression may provide some extra prognostic information of certain clinical significance in TNBC. For example, compared to untreated primary breast cancer, BCAT1 mRNA levels were elevated in tamoxifen-resistant breast cancer samples, and an increased level of BCAT1 expression was associated with an unfavourable prognosis in antioestrogen-resistant breast cancer [38]. This clinical application requires further validation in larger trials, but the potential is promising, as observed for CD133. In addition, as a heterogeneous disease, TNBC is suggested to be further classified into seven distinct molecular subtypes using gene expression profiling, which leads to different survival benefits from NAC and other biomarker expression patterns [42]. As shown by the bioinformatical analysis in this study, high BCAT1 expression is correlated with unfavourable responses to NAC for patients with TNBC, which unfortunately, was not validated with a larger collection of datasets. The expression patterns of BCAT1 and CD133 among multiple TNBC subtypes were not evidently distinguished. This may be due to limited information of the public data on unspecific responses to NAC, which may have blurred out the statistical significance. However, our findings suggested the potential of BCAT1 on predicting the NAC benefit, and subsequent clinical trials were warranted. Furthermore, this study identified a high-BCAT1- and high-CD133-expression subset of patients with TNBC, who had significantly worse prognoses in the cases of both basal-like and non-basal-like tumours. This subset of patients may have specific molecular subtypes, which will be tested in further genetic profiling studies. Additionally, clinical trials are warranted to explore the predictive significance of BCAT1 and CD133 expression patterns in patients who may benefit more from neoadjuvant chemotherapies. Thus, the combined biomarker panel showed certain prognostic importance in TNBC with the potential for pathological evaluation and clinical application.

Tumour-infiltrating lymphocytes (TILs) are another emerging indicator of TNBC prognosis. Studies have suggested that TILs can serve as a positive prognostic biomarker in TNBC but not in the luminal subtypes, as first reported in the BIG 2–98 trial and subsequently confirmed in two independent phase III adjuvant randomised trials (United States Eastern Cooperative Oncology Group trials 2197 and 1199) [43, 44]. Detection of more stromal TILs at diagnosis indicates a better outcome after adjuvant anthracycline-based chemotherapy, and the results supporting the prognostic value of TILs in TNBC are considered level I evidence [45–47]. Consistent with the results of these studies, the prognostic significance of TILs was also demonstrated in this study. We observed a lower level of BCAT1 expression in the high-TIL group, which may have reflected an immune reaction and suppression of tumour progression, leading to a favourable prognosis in patients with TNBC. Similarly, a lower level of CD133 expression was detected in the high-TIL group, which may be associated with higher levels of tumour immune susceptibility. However, further studies are needed to reveal the underlying mechanisms involved in complex interactions between the tumour and body immunity.

The strengths of this study include the large sample size, a long follow-up period, and extensive statistical analysis of the data. Regarding study limitations, an even larger cohort study, which is currently underway, is necessary to validate the clinical application of the investigated biomarkers. Furthermore, to better understand the underlying mechanisms, animal experiments involving BCAT1 and CD133 analysis and studies using genetically engineered TNBC cell lines are also warranted. Currently, BCAT1 is considered as a promising drug target for anticancer therapy, and small-molecule inhibitors of this enzyme are being developed.

## Conclusions

In this study, BCAT1 and CD133 were shown to be independent prognostic biomarkers for TNBC. Further studies are warranted to validate the potential clinical applications of these biomarkers and BCAT1- or CD133-targeted therapies for TNBC.

## Data Availability

All supporting data and materials are available from the corresponding author upon reasonable request.

## Abbreviations

BCAT1: Branched-chain amino acid transferase 1
BLBC: Basal-like breast cancer
CSC: Cancer stem cells
DFS: Disease-free survival
ER: Oestrogen receptor
H&E: Haematoxylin and eosin
HER2: Human epidermal growth factor receptor 2
IHC: Immunohistochemical
OS: Overall survival
pCR: pathological complete response
PR: Progesterone receptor
ROC: Receiver operating characteristic
SE: Standard error
TIL: Tumour-infiltrating lymphocytes
TMA: Tissue microarray
TNBC: Triple-negative breast cancer
NAC: Neoadjuvant chemotherapy
